# Assessment of early onset surface damage from accelerated disinfection protocol

**DOI:** 10.1186/s13756-019-0467-9

**Published:** 2019-01-31

**Authors:** Hyungyung Jo, Alyssa M. West, Peter J. Teska, Haley F. Oliver, John A. Howarter

**Affiliations:** 1 School of Materials Engineering, Purdue University, 701 W. Stadium Avenue, West Lafayette, IN 47907 USA; 2 Department of Food Science, Purdue University, 745 Agriculture Mall Drive, West Lafayette, IN 47907 USA; 3 Diversey Inc, Charlotte, NC 28273 USA; 4 Environmental & Ecological Engineering, Purdue University, 701 W. Stadium Avenue, West Lafayette, IN 47907 USA

**Keywords:** Surface damage, Roughness, Contact angle, Disinfectant, Material testing

## Abstract

**Background:**

The objective of this study was to evaluate the extent and potential mechanisms of early onset surface damage from simulated wiping typical of six-months of routine disinfection and to assess the subsequent microbial risk of surfaces damaged by disinfectants.

**Methods:**

Eight common material surfaces were exposed to three disinfectants and a neutral cleaner (neutral cleaner, quaternary ammonium, hydrogen peroxide, sodium hypochlorite) in accelerated aging tests to simulate a long-term disinfection routine. Materials were also immersed in dilute and concentrated chemical solutions to induce surface damage. Surfaces were chemically and physically characterized to determine extent of surface damage. Bactericidal efficacy testing was performed on the Quat-based disinfectant using a modified version of EPA standard operating procedure MB-25-02.

**Results:**

The wiping protocol increased surface roughness for some material surfaces due to mechanical abrasion of the wiping cloth. The increased roughness did not correlate with changes in bactericidal efficacy. Chemical damage was observed for some surface-disinfectant combinations. The greatest observed effects from disinfectant exposure was in changes in wettability or water contact angle.

**Conclusions:**

Early onset surface damage was observed in chemical and physical characterization methods. These high-throughput material measurement methods were effective at assessing nanoscale disinfectant-surface compatibility which may go undetected though routine macroscale testing.

## Background

Environmental cleaning practices are key to preventing the transmission of healthcare-associated infections (HAIs), which resulted in ~ 75,000 deaths in the United States in 2011 [1]. Surface-applied disinfectants are frequently used in environmental cleaning as part of the regular hygiene plan. The high-frequency of disinfectant products coming into contact with a wide range of surfaces has the potential to induce incremental damage through each exposure. The risk of damage to the surface is subject to several influences. The age of the surface, exposure time, chemical composition of the disinfectant, and method of disinfectant application all contribute to potential surface damage [2]. The CDC’s Guidelines for Disinfection and Sterilization in Healthcare Facilities lists the shortcomings of various disinfection chemicals, including what kinds of surfaces they can damage [3].

An article by Spaulding in 1964 reviewed alcohol as a disinfectant and showed that alcohol has been known to damage certain materials [4]. Likewise, disinfectant-induced corrosion of stainless steel has been reported where the damage is undetectable to the naked eye [2]. Although small, this mild degree of surface damage can provide a place for bacteria to inhabit and grow. Repeated use of a disinfectant on a damaged surface will only exacerbate the damage and create a wider berth for bacteria to inhabit [2]. Although it has been known for some time that repeated exposure of a surface to a disinfectant can cause surface damage, the effect on disinfectant efficacy has not been quantified. The objective of this study was to evaluate the extent and potential mechanisms of early onset surface damage from simulated wiping typical of six-months of routine disinfection and to perform a limited assessment of the subsequent microbial risk of surfaces damaged by disinfectants. We hypothesized that subtle changes in the surface chemistry or morphology, as a result of disinfection-induced damage will create potential micro-environments where bacterial pathogens can persist.

## Methods

### Surface damage characterization

Six different types of polymers, a glass, and a metal sample are examined. The six polymers are high density polyethylene (HDPE), acrylonitrile butadiene styrene (ABS), ethylene propylene diene monomer (M-class) rubber (EPDM), low density polyethylene (LDPE), Formica-like material (Garolite LE, McMaster-Carr), and polycarbonate (PC). Table 1 shows their mechanical property (hardness) and water absorption property, which were provided by the distributor, McMaster Carr. These six polymer surfaces were selected as they represent a range of hardness and mechanical strength. We intentionally selected polymers that were not manufactured into specific products to focus on the material-disinfectant compatibility for common polymer materials. Microscope slide glass and 304 stainless steel were used for the glass samples, and metal samples, respectively. All polymer and metal samples were cut into coupons (1 ft. × 2 in.) used for the control, wiping, and immersing by being exposed to disinfectants which were tested at both ‘full strength’ and diluted per label specifications. The coupons were subsequently cut into approximately 1 in × 2 in samples for characterization. The Quaternary (Quat) disinfectant (Virex II 256, EPA Registration 70,627–24, Diversey Inc., Charlotte NC) was utilized as full strength (no dilution), and diluted solution at 1:256 with deionized water. The Improved Hydrogen Peroxide disinfectant (Oxivir Five 16, EPA registration 70,627–58, Diversey Inc., Charlotte NC) was used full strength and diluted at 1:16 with deionized water. The sodium hypochlorite disinfectant (Clorox Germicidal Bleach, EPA registration 5813–100, Clorox Company, Pleasanton CA) was utilized full strength and diluted at 1:8 with deionized water, as a control, Prominence Neutral Cleaner (Diversey Inc., Charlotte NC) was used full strength and at a 1:256 dilution.

**Table 1:** Summary of materials with hardness and water absorption; data provided by manufacturer

Material	Hardness (Hardness rating)	Water absorption
HDPE	Durometer 60D (Medium)	Not rated
ABS	Rockwell R100 (Hard)	0.65%
EPDM	Durometer 40A (Medium soft)	Not rated
LDPE	Durometer 40D (Medium soft)	Not Rated
Formica	Rockwell M100 (Extra hard)	1.20%
PC	Rockwell R120 (Hard)	0.25

All utilized solutions are summarized in Table 2. One set of samples was wiped twice in each direction as consistent as possible with disposable Kimtech wipes wetted with a predetermined amount of liquid and allowed to dry for 10 min. The wiping compression stress was approximately 0.04 MPa, applied by hand to simulate “real” cleaning conditions. This cycle was repeated 200 times for all of the product/surface combinations. Another set of samples were immersed in closed containers with the disinfectant solutions for 4 weeks at room temperature. All treated samples were rinsed with deionized water before being characterizing. The specimen which were wiped 200 times at label-specified dilutions were intended to mimic 6 months of routine disinfection. Protocols for immersed and off-label concentrations were intended to mimic an aggressive “worst-case” for chemical surface damage or material incompatibility. Notably, only the wiped samples exposed at the label specified dilution is truly mimicking the real use case. However, we hypothesize that by having specimen continuously immersed in the disinfectant solution and also wiped under concentrated (i.e. ‘full strength’) conditions, we are able to potentially induce accelerated chemical damage which can reveal antagonistic material-disinfectant combinations not detected by the diluted-wiping test protocol. It may be possible to use the accelerated ‘off-label’ protocol for rapid laboratory-based screening of material-disinfectant compatibility.

**Table 2:** Summary of four solutions with product names and dilution ratio

	Solution	Product	Dilution	pH (full strength)	pH (diluted)
a	Neutral cleaner	Diversey Prominence	1:64	8.4	6.8
b	Quat disinfectant	Diversey Virex II 256	1:256	10.2	8.8
c	Hydrogen peroxide disinfectant	Diversey Oxivir Five 16 concentrate	1:16	1.0	1.9
d	Chlorine disinfectant	Clorox bleach	1:8	12.5	10.4

A conventional goniometer was used to measure contact angles. A sessile drop of 4 μL was supplied to measure contact angle between the sample and the droplet. The samples were also characterized by Fourier transform infrared (FTIR) spectroscopy in order to identify bonding changes before and after wiping and immersing. Samples were weighed before and after immersion tests to determine water absorption. Atomic Force Microscopy (AFM) was utilized to obtain the topography of damaged surfaces in a tapping mode of operation. Surface topography was carried out with measuring surface roughness (R_q_). X-ray photoelectron spectra (XPS) were obtained using Kratos Axis Ultra DLD spectrometers with Al Kα radiation (*hν* = 1486.58 eV).

### Bactericidal efficacy testing

Bactericidal efficacy testing was only performed on the diluted Quat disinfectant using a modified version of EPA standard operating procedure MB-25-02 [EPA]. Briefly, bacterial culture was mixed with a soil load (yeast, mucin, and BSA) and inoculated onto 1“× 1” coupons of the polycarbonate, low-density polyethylene, and Formica materials. The coupons were desiccated for 1 h to adhere the bacteria to the surface. The disinfectant was then applied to the coupons and left to sit for the label contact time. After the contact time was reached, 10 mL of neutralizing buffer was added, and the coupon was vortexed in the neutralizing buffer. The solution was vacuum-filtered onto a filter membrane to recover any bacteria that were left. The membrane filter was plated onto TSA for 24–48 h. at 37 °C, and colonies were counted. This procedure was repeated for the treated surfaces and undamaged samples (which established a baseline of disinfectant efficacy).

The Quat-based disinfectant used was Virex II 256. The concentrated disinfectant was diluted at 1:256 using hard water, following EPA MB-25-02 [5]. The disinfectant label contact time was 10 min, and five biological replicates were done for each surface-disinfectant combination. The bacteria tested was *Staphylococcus aureus* (ATCC #6538), the standard test microbe used in EPA MB-25-02 [5].

### Statistical analysis

Statistical Analysis Software (SAS), version 9.4, was used to perform analysis of the data. All data were transformed to log10 reduction values for analysis. One-way ANOVA with Tukey Honest Significant Difference (HSD) test was used to determine if differences in disinfectant efficacy existed between the three surface treatments (α = 0.05).

## Results

Table 3 shows the summarized results of the contact angle, FTIR, and optical microscopy measurements after the wiping and immersing tests. None of the tested samples exhibited significant mass changes as a result of immersion tests. The only changes in the results are marked with F and D, which indicate full strength solution and diluted solution, respectively. A bullet point means no change. For contact angle data, increased and decreased contact angles after treatment are indicated with arrows. Each section marked with red boxes is discussed in detail in the following section.

**Table 3:**
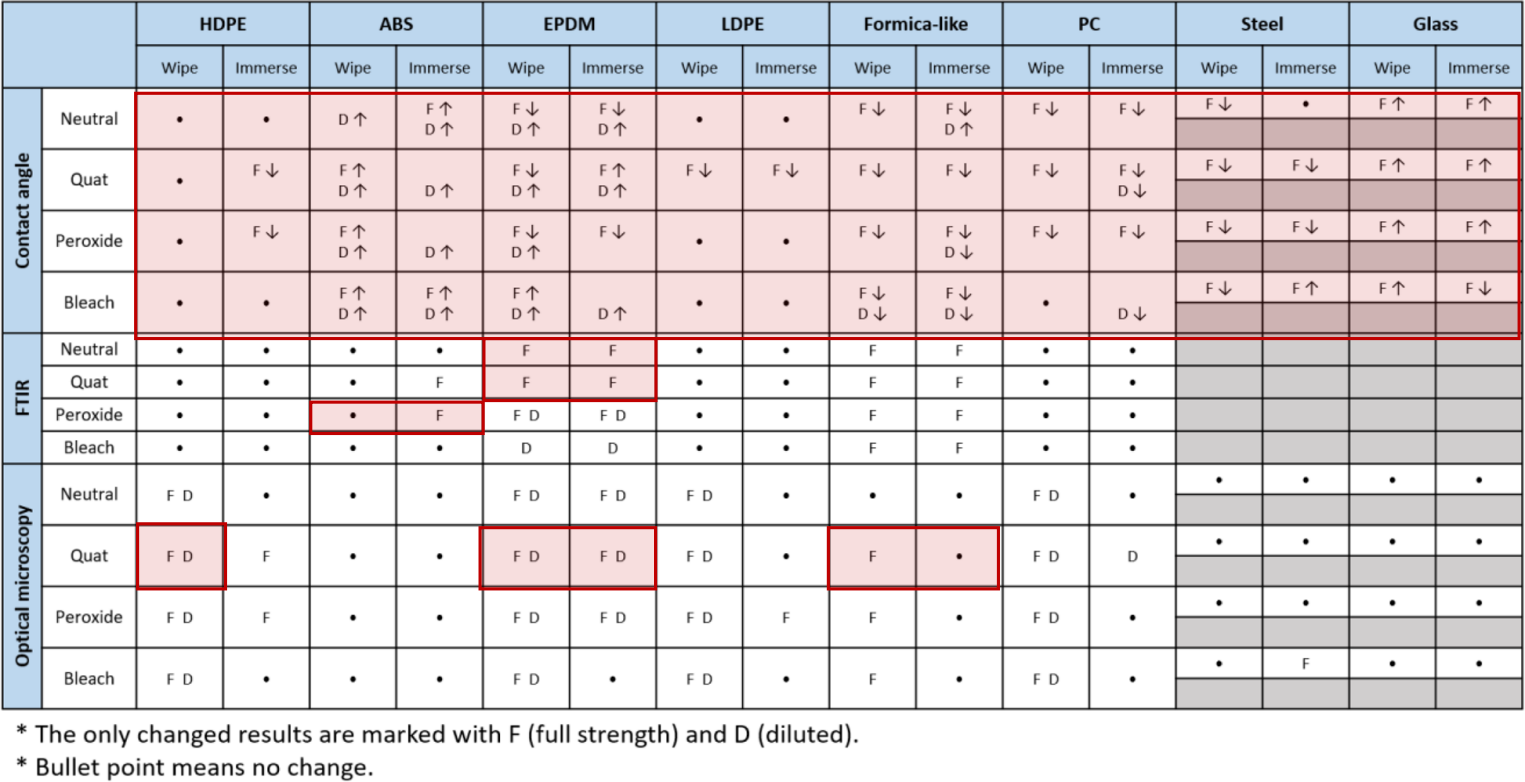
Summarized wiping and immersing test results with full strength and diluted solutions on the six polymers, steel, and glass

**Fig. 1:**
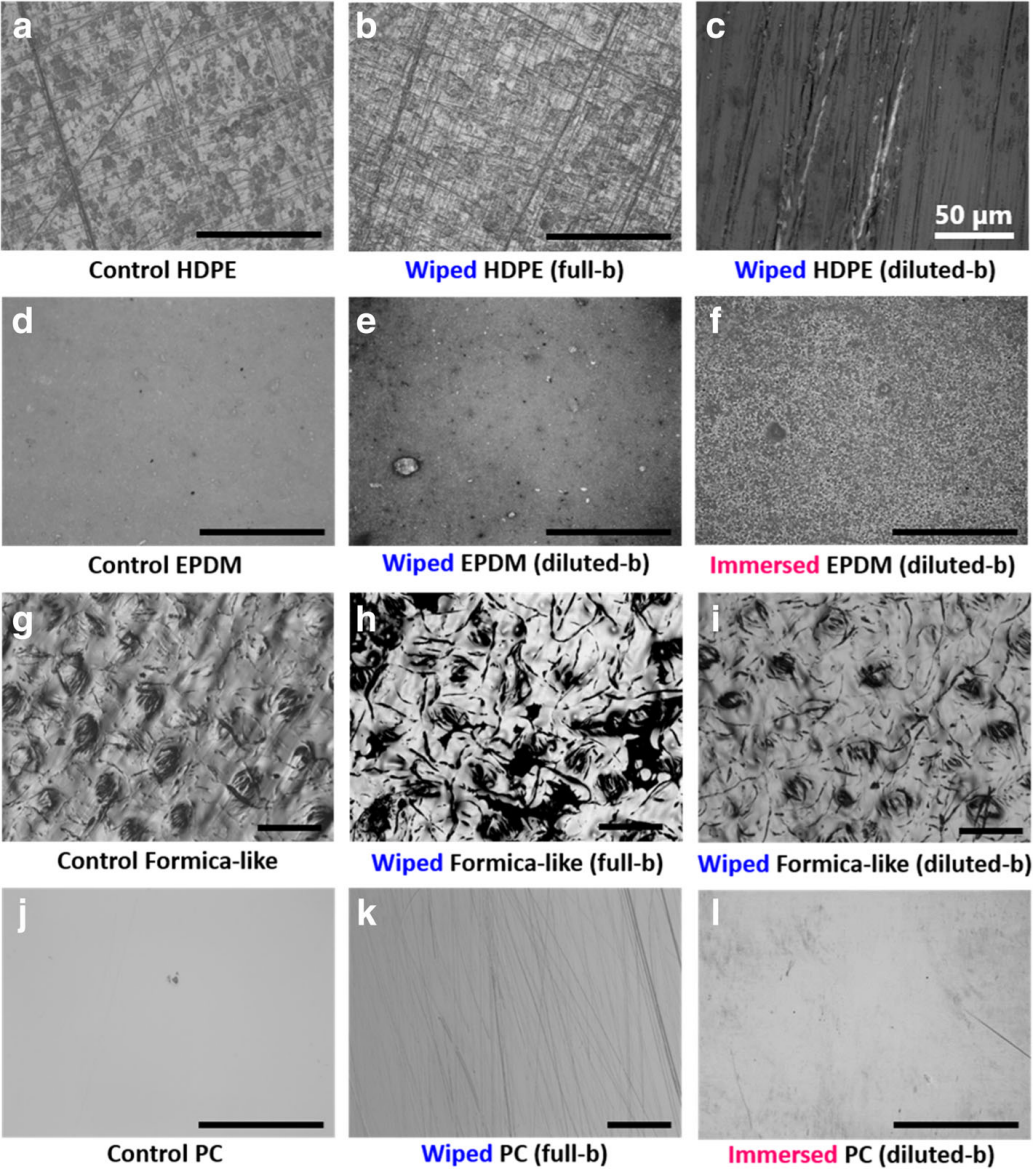
Optical images of HDPE, EPDM, Formica-like surface, and PC (black scale bar shows 500 μm)

Figure 1 shows optical micrographs of four control samples and their changed surfaces after wiping and immersing. Wiped HDPE has increased directional scratches, although the control HDPE had some scratches originally. Regardless of the kind of solution, most wiped HDPE surfaces show stress whitening along the deep scratches, as seen in Fig. 1(c). On the other hand, optical images of EPDM show a significant chemical effect. Wiping did not generate scratches mechanically because EPDM is a very compliant elastomer, the roughened surface seems to be the result of chemical damage. Immersed EPDM especially shows a chemically etched surface with greatly increased roughness, even from the diluted disinfectant as shown in Fig. 1(f). The control Formica surfaces exhibit a wavy texture with uniform porous bumps. Wiping with full strength disinfectant could have produced a compressed wavy texture with some grooved defects, as shown in Fig. 1(h). However, the use of diluted disinfectant did not make severe mechanical damage on the Formica surface, comparatively. Regarding the PC, while the control PC has an almost flawless surface, the wiped PC surface exhibited scratching as shown in Fig. 1(k). Additionally, immersed PC in diluted Quat disinfectant also showed some chemical damage on the surface.

**Fig. 2:**
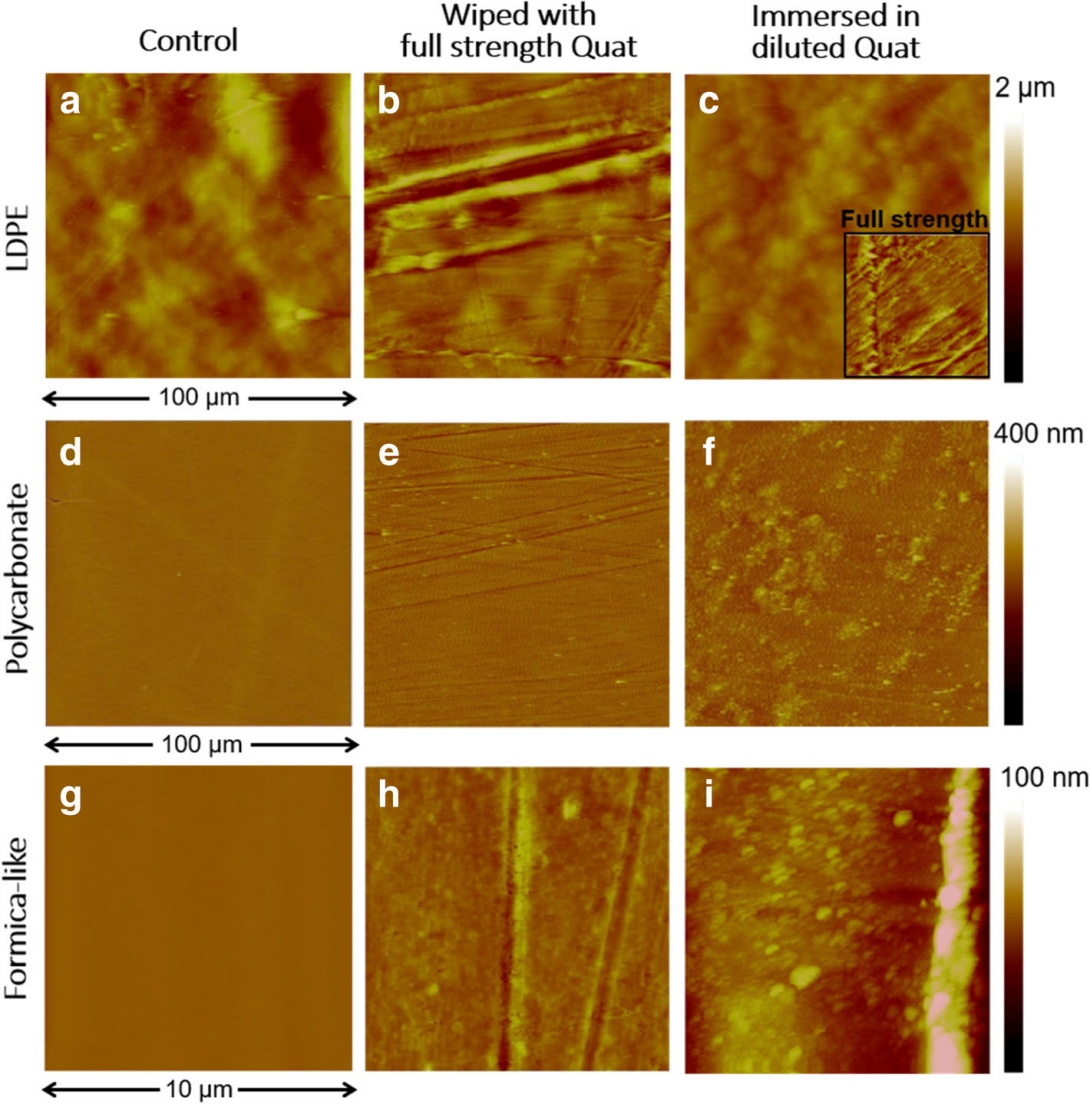
AFM images of LDPE, PC, and Formica-like surface as control (**a**, **d**, **g**), wiped with full-strength Quat disinfectant (**b**, **e**, **h**), and immersed in diluted Quat disinfectant (**c**, **f**, **i**). The lower-right corner of the immersed-LDPE image (**c**) shows the effects of the use of full-strength Quat. Scan sizes of the LDPE and PC images are 100 μm, and the Formica-like surface images are 10 μm

Micro-scale surface damage was selectively examined by atomic force microscopy (AFM) operated in a tapping mode to minimize surface damage caused by scanning. The surfaces for samples wiped with full-strength Quat disinfectant are shown in the middle column, and samples immersed in diluted Quat are shown in the right column in Fig. 2, with the control samples in the left column. The scan sizes of LDPE and PC are 100 μm while, the Formica surface scan size is 10 μm which was necessary to avoid the large surface features (bumps visible in Fig. 1g) which are incompatible with the AFM scanning tip. The AFM images of samples are shown with various color scales: a 2 μm color scale for LDPE, 400 nm for PC, and 100 nm for the Formica sample. The scales were chosen depending on the original roughness of the samples and the scan size.

Table 4 presents the summary of the roughness quantification for LDPE, PC and Formica-like surfaces. For LDPE wiped with diluted disinfectant, the surface roughness decreased to 167 nm because of the repeated wiping process on the ductile LDPE surface. However, Fig. 2(b) shows a deep trench parallel to the wiping direction, representing an increased roughness of 330 nm. Also, LDPE immersed in diluted Quat disinfectant showed insignificant chemical damage on its surface, while the use of full-strength Quat disinfectant etched the LDPE surface, as shown in Fig. 2(c). Surface-roughness values demonstrated this chemical effect quantitatively, with values of 163 nm and 342 nm resulting from the use of diluted and full-strength disinfectant, respectively. When the scan size was 100 μm, the control PC showed 13.5 nm surface roughness as shown in Fig. 2(d). Although the AFM image of the wiped PC shows some shallow scratches, surface roughness was changed only insignificantly to 15.7 nm. The AFM image of PC immersed in diluted Quat disinfectant shows that the chemically affected PC surface had tens of nanometers of sticky residue. In terms of Formica, the flat surface between pores was scanned at 10 μm to investigate microscale damage since the original features of the Formica surface are too large to scan in AFM. The wiping process produced some scratches, and the scratched surface changed to appear slightly mottled as shown in Fig. 2(h). Additionally, immersed Formica had a few micrometers of small particles which were chemically damaged while immersing in disinfectant.

**Table 4:**
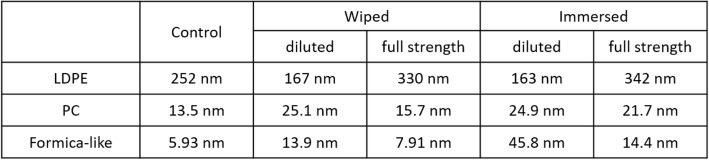
Roughness values from the AFM results. Scan sizes of the LDPE and PC images are 100 μm, and the Formica-like surface images are 10 μm

**Fig. 3:**
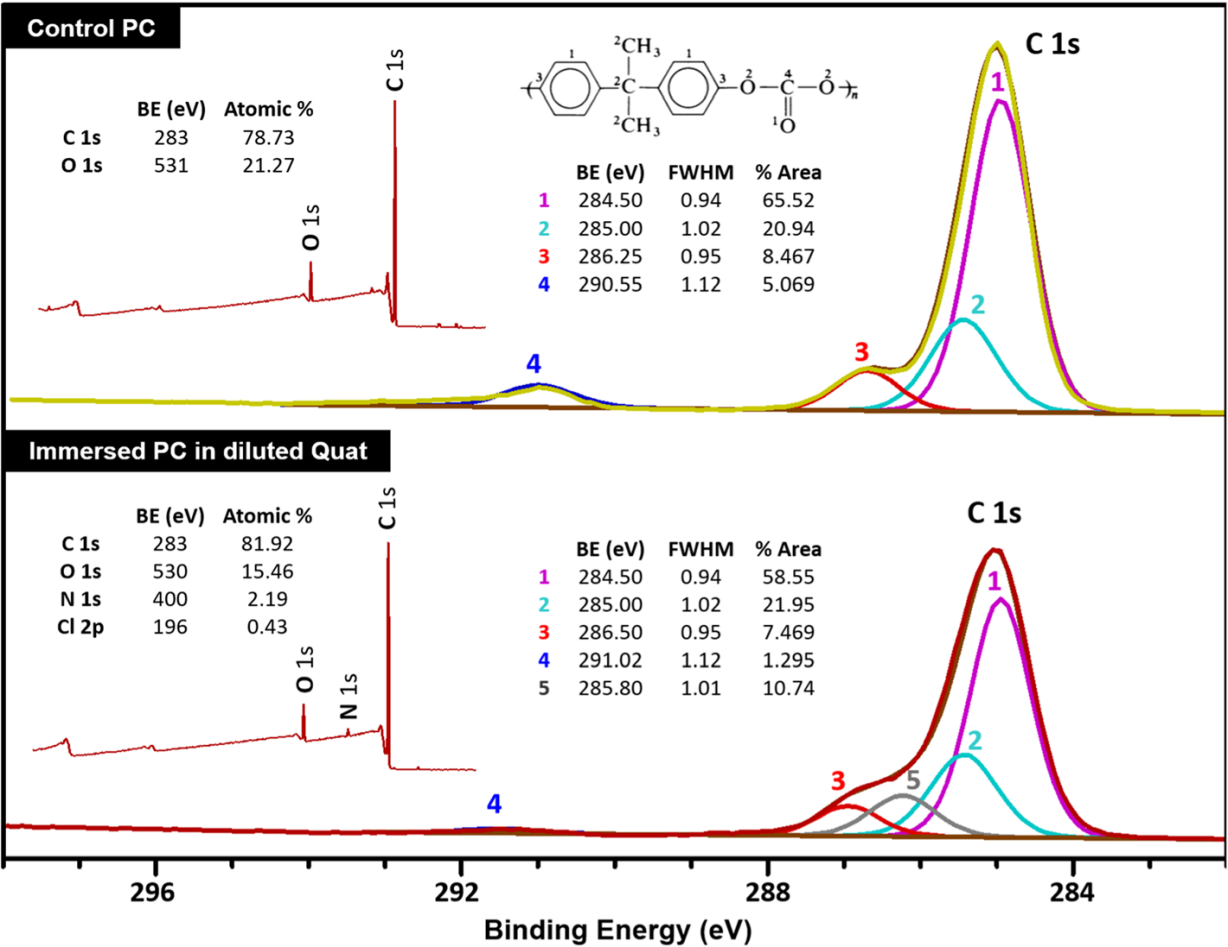
Wide scan (inside-left) and C 1 s (right) fitted with XPS spectra of control (above) and immersed (below) PC in diluted Quat disinfectant

Figure 3 shows the quantification tables with the XPS spectra of control and immersed PC in diluted Quat disinfectant. Although the immersed PC in diluted Quat disinfectant had sticky residues, which are shown in the AFM image and optical image (see Fig. 2). Atomic concentration of nitrogen and chlorine on the immersed PC were measured using XPS. Also, the C 1 s peak of the control PC was deconvoluted into four species as shown in the formula in Fig. 3. Here the four different carbon groups appear in the molecular structure. (from numbers 1 to 4 in the formula due to different carbon groups presenting in PC as shown in the molecular structure in Fig. 3.) The % areas of each carbon calculated from C 1 s spectra of control PC are roughly matching for 10:3:2:1 which is the ratio of each number of different carbons in ideal PC. After immersing for 4 weeks, number 4 peak for C=O bonds on immersed PC was significantly decreased. Also, the measured C-O and C=O bonds were slightly shifted to higher binding energy, indicating changes in chemical bonding.

**Fig. 4:**
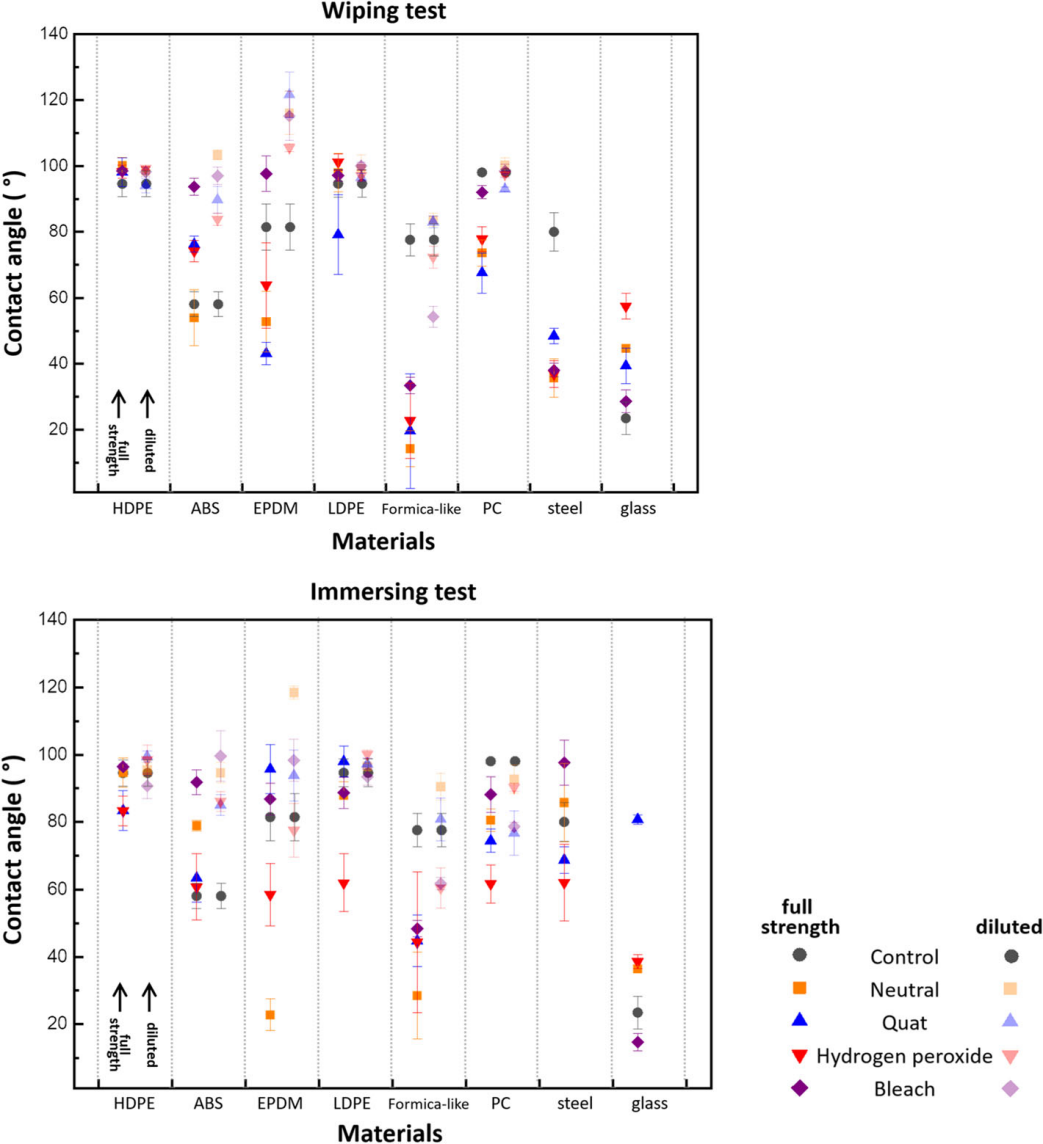
Contact angles of materials before and after wiping and immersing tests with full strength and diluted solutions

Figure 4 presents static water contact angle measurements for the eight surfaces exposed to wiping and immersion tests. Notably, HDPE and LDPE show the least overall variation in contact angle as a result of exposure to the disinfectants. Most other surfaces show a general trend of a decrease in contact angle after exposure, with the exception of ABS which exhibited increased contact angles overall and EPDM which exhibited both increased and decreased contact angles depending on the concentration and disinfectant chemistry. The contact angle test itself is a combined measure of the surface roughness and surface chemistry as both factors affect the apparent contact angle for a material. For this reason, it is a very effective method for quickly screening for surface damage, and here we can broadly interpret changes observed in the immersion test as either chemical attack on the surface or the absorption and swelling of the material.

**Fig. 5:**
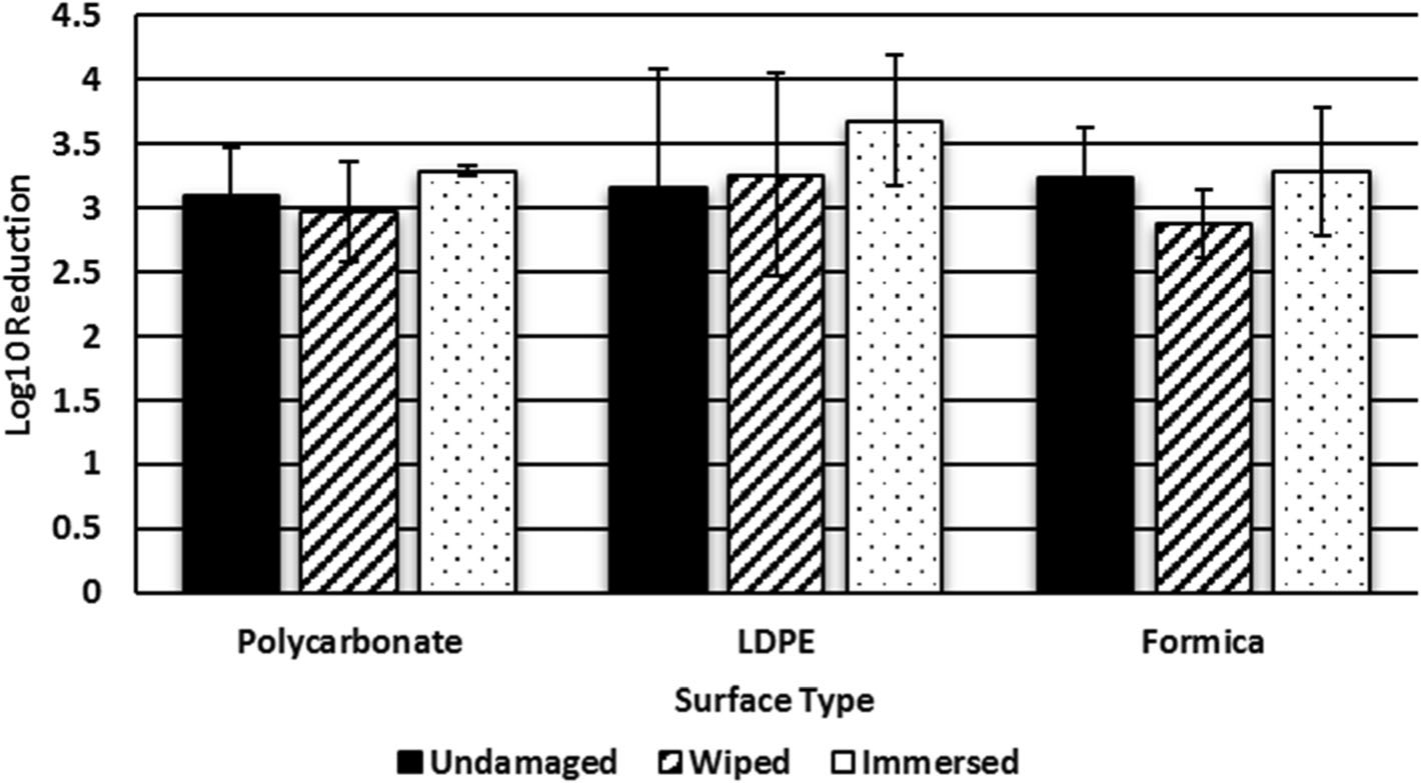
Log10 reduction values of *S. aureus* for Virex II 256 (Quat-based disinfectant) on three polymer surfaces

Figure 5 presents Log10 reduction for polycarbonate, LDPE and Formica surfaces exposed to *S. aureus* and disinfected with Virex II 256 following a modified version of EPA standard operating procedure MB-25-02 [5]. On average, the disinfectant achieved a 3.16 log10 reduction on the undamaged surfaces. Specifically, Virex II 256 achieved an average 3.26 log10 reduction on wiped LDPE and an average 3.68 log10 reduction on immersed LDPE. Statistical analysis determined that there was not a significant difference in bactericidal efficacy between the undamaged, wiped, and immersed LDPE surfaces (*P* > 0.05). Virex II 256 achieved an average 2.88 log10 reduction on wiped Formica and an average 3.29 log10 reduction on immersed Formica. There was no significant difference in disinfectant efficacy between the undamaged, wiped, and immersed Formica surfaces (P > 0.05). Virex II 256 achieved an average 2.97 log10 reduction on wiped polycarbonate and an average 3.29 log10 reduction on immersed polycarbonate.

## Discussion

### Mechanical damage characterization

In order to characterize microstructure, optical microscopy was initially utilized to analyze overall surface damages on treated surfaces because it is expedient and does not require special sample preparation. Stress whitening was observed at high magnification of optical microscopy, especially on wiped HDPE and wiped LDPE. Stress whitening is a general feature of mechanical surface damage induced by tensile deformation on various polymeric materials and is aesthetically undesirable [6, 7]. Since LDPE has a lower scratch resistance than HDPE, the LDPE showed more scratching during the wiping process. Optical images of HDPE and LDPE were helpful for detecting mechanical damage, while at the microscale chemical damage was hardly observed. In contrast, optical images of EPDM showed a significant chemical effect since the degradation of EPDM can take place in specific chemical environments, as when the disinfectant contains hydrocarbons, hypochlorite, or peroxide [8]. Regarding Formica, this surface seemed to be vulnerable to mechanical impact from wiping disinfection, especially with full-strength disinfectant. This result occurred because full strength disinfectant leaves a residue which evaporates with difficulty, while diluted disinfectant evaporates quickly. Furthermore, since the Formica surface can absorb solution into its laminated layers, the residue of full-strength disinfectant can act more aggressively. Therefore, the repeated wiping of its surface soaked with full strength disinfectant could easily result in mechanical damage. With respect to PC, it has a high impact strength but relatively low scratch resistance [9]. Since PC is transparent it is largely used on clear, see-through surfaces. Fortunately, the mechanical damaging on PC was not aggressive enough to affect the macroscopic transparency, which did not significantly differ from the effects of various concentrations of disinfectant. However, it is noteworthy that even after 4 weeks, when diluted Quat disinfectant was utilized as an immersing solution, immersed PC created a sticky surface which was difficult to remove when rinsed with DI water and was more excessive than the residues deposited on other surfaces from the disinfectant solutions.

Since AFM is able to measure the length and depth of surface damages with a surface profile, it could be a powerful quantitative method tool for surface damage characterization. Despite the use of gentle textured Kimtech wipes, wiped LDPE showed various sizes of scratches on the surface, including one very deep trench, a scratch with a depth of 925 nm as shown in Fig. 2(b). Important to note, bacteria could grow in this space since the sizes of common bacteria are approximately 0.5-1 μm [10]. Furthermore, the scratch with built-up side ridges could lower cleanability: frequent wiping of a ductile surface could produce scratches capable of retaining bacteria. Even though wiping PC also creates a scratched surface, the increased roughness was only a few nanometers, which would not be large enough to allow bacterial growth. From the AFM images of Formica, when the Formica sample was treated with disinfectant, the compressed cellulose fibers that absorbed the solution emerged as small particles on the immersed surface as shown in Fig. 2(i). Judging from the optical images, significant mechanical damage appeared on the Formica surface, however, the AFM results also showed that at the nanoscale, the immersing process resulted in a chemically changed Formica surface.

### Chemical damage characterization

Some polymers can absorb small molecules, such as water molecules, as shown in Table 1. Thus, absorption of solutions could be a major chemical impact when samples were immersed in disinfectants [11, 12]. In particular, it is well known that ABS has low chemical resistance to oxidizing agents, which are available to break up the ABS chains [13]. The FTIR results of immersed ABS show a modified IR spectrum with decreased intensities when full strength hydrogen peroxide disinfectant was utilized. These findings may indicate a partial scission of bonds in the ABS, causing decreases in chemical bonds. Aqueous hydrogen peroxide can be dissociated to a free radical, a strong oxidizing agent that can damage substrates [14]. Thus, the higher the concentration of hydrogen peroxide disinfectant, the more aggressive the solution can be on polymer surfaces. Looking at Table 3, the Formica-like surface treated with full strength solutions is also marked as “changed”. Since the Formica surface has a laminated structure, it was able to adsorb solutions physically, which demonstrated a marked change due to its dark surface color. For the immersed PC in diluted Quat disinfectant, sticky residues that were difficult to remove were formed, but the FTIR did not show any notable changes. Since the ATR-FTIR penetration depth is 2 μm [15], the measurement is not sensitive to the outermost surface of the material. Thus, even though FTIR did not show any changes, it is possible that nanometer-scaled damage occurred on the surface; the damaged PC surface chemistry was measured using XPS.

Regarding the results of XPS, the double bonds that were opened to make covalent bonds with nitrogen from the diluted Quat disinfectant could have precipitated the decreased ratio of C=O. Binding energy also provided both elemental and chemical information. The measured C-O and C=O bonds were slightly shifted to a higher binding energy, thus, it is possible that covalent bonds with nitrogen adsorbed from the diluted Quat disinfectant [16]. Additionally, the ratios of carbon single bonds with hydrogen which is the number 2 carbon in formula increased when the ratio of C=O bonds was significantly decreased. It is also noticeable that no sticky residue occurred with the use of full strength Quat disinfectant. This result indicates that the diluent (water) likely plays a significant role in enabling the specific degradation mechanism when Quat disinfectant and PC are combined and that the residue on the dilute Quat-PC surface is not simply material deposited from the disinfectant [17]. Subsequent XPS data confirmed chemical changes at the PC surface [18].

### Bactericidal efficacy

The EPA testing method used in this study determined that there was not a significant difference in bactericidal efficacy between undamaged, wiped, and immersed samples. This might be because even undamaged surfaces already had a lot of scratches or the scratches might not have been significantly different to provide protection from disinfection in one cased but not the other. Although LDPE have been observed as mechanically susceptible surfaces from microstructure characterization, there might not be measurable differences from those in the bactericidal efficacy test. The Formica surface also originally had a wavy and bumpy texture, thus, the mechanical damages from the disinfectant process might not have affected the bactericidal efficacy among the undamaged, wiped, and immersed Formica surfaces. Regarding the PC, while PC surface was chemically damaged from the immersing process, it might not be comparable to differentiating disinfectant efficacy between undamaged and immersed PC surfaces. Therefore, the EPA testing method used in this study may not be sensitive enough to detect the differences in disinfectant efficacy due to surface damage. We feel that further study is warranted with respect to disinfectant efficacy of lightly mechanically damaged surfaces as observed from the gentle wiping protocol in this study to better understand the critical threshold of surface damage which can result in loss of bactericidal efficacy.

## Conclusion

Surfaces which experience a high-frequency of exposure to disinfectant chemicals may be at additional risk of critical surface damage that renders the surface more challenging to disinfect. Chemical compatibility tests and other screening protocols may overlook the effects of long-term exposure and may otherwise be insufficiently sensitive to changes at the outermost interface material. The surface characterization of eight surfaces exposed to four different disinfectants was used to assess the sensitivity of detection methods for early onset surface damage. The surfaces tested were lightly damaged from a test protocol simulating six-months of routine disinfection. As such, widespread macroscopic damage was not observed. Although full strength disinfectants are not usually utilized in practice, the test was able to demonstrate chemical resistance under an aggressive condition. Some chemical damage was detected using water contact angle and XPS, whereas more conventional FTIR spectroscopy did not detect significant chemical changes. The discrepancy here is attributed to the surface sensitivity of the respective techniques. Most treated surfaces showed no significant chemical damage for any disinfectant exposure (including the concentrated disinfectants under immersed conditions), this further emphasizes the impact of mechanical abrasion as a key source of critical surface damage. Bactericidal efficacy of Virex II 256 (Quat-based) was assessed for select surfaces which exhibited moderate surface damage. However, the EPA testing method used in this study did not detect significant differences in disinfectant efficacy due to modest surface damage. Further study is warranted for surfaces that have experienced more aggressive surface damage, possibly from real in-service material samples, to determine a critical defect population necessary to alter bactericidal efficacy.

## Abbreviations

ABS: Acrylonitrile Butadiene Styrene
AFM: Atomic force microscopy
EPDM: Ethylene Propylene Diene Monomer (M-class) rubber
FTIR: Fourier transform infrared
HDPE: High density polyethylene
LDPE: Low density polyethylene
PC: Polycarbonate
Quat: Quaternary Ammonium Compounds
XPS: X-ray photoelectron spectra
